# Effect of Mn-Doped ZnFe_2_O_4_ Ferrites on Structural Changes and Magneto-Optical Behavior in Nematic Liquid Crystals

**DOI:** 10.3390/ma18245660

**Published:** 2025-12-17

**Authors:** Peter Bury, Marek Veveričík, František Černobila, Hima Patel, Ramesh V. Upadhyay, Kinnari Parekh, Veronika Lacková, Michal Rajnak, Ivo Šafařík, Koryun Oganesyan, Milan Timko, Peter Kopčanský

**Affiliations:** 1 Department of Physics, Žilina University, Univerzitná 1, 010 26 Žilina, Slovakia; 2 Dr. K C Patel R & D Centre, Charotar University of Science and Technology, Changa, Anand 388421, Gujarat, Indiarvu.as@charusat.ac.in (R.V.U.); kinnariparekh.rnd@charusat.ac.in (K.P.); 3 Institute of Experimental Physics, SAS, Watsonova 47, 040 01 Košice, Slovakiaoganesyan@saske.sk (K.O.);; 4 Department of Nanobiotechnology, ISBB, Biology Centre, CAS, Na Sádkách 7, 370 05 České Budějovice, Czech Republic; 5 Regional Centre of Advanced Technologies and Materials, Czech Advanced Technology and Research Institute, Palacký University, Šlechtitelů 27, 783 71 Olomouc, Czech Republic; 6 A.I. Alikhanyan National Science Lab, Yerevan Physics Institute, Alikhanyan Br.2, Yerevan 0036, Armenia

**Keywords:** nematic liquid crystals, Mn-doped zinc ferrite nanoparticles, magnetic properties, acoustic attenuation, magneto-optical effect

## Abstract

The effect of Mn-doped zinc ferrite nanoparticles at a low volume concentration (1 × 10^−4^) on structural changes in the nematic liquid crystals 6CHBT and 5CB, induced by weak magnetic fields, was investigated using surface acoustic wave (SAW) and light transmission (LT) techniques. Structural changes caused by the applied magnetic field, in both increasing and decreasing modes, as well as after pulsed changes, were examined by measuring the responses of SAW attenuation and LT using a linearly polarized laser beam. The influence of nanoparticle shape (rods, needles, and clusters) and temperature on the structural changes was investigated. A shift in the threshold field and the transition temperature was observed. In addition, the magnetic properties of the individual samples in powder form were examined using M–H curves, M–T curves, and XRD patterns. The results obtained from all measurements are compared, and the effectiveness of each technique, considering the influence of nanoparticle shape and suspension stability, was evaluated.

## 1. Introduction

Nematic liquid crystals (NLCs), characterized by the orientational order of their molecular axes along a preferred direction, represent, in some materials, a state of matter between the crystalline state and the isotropic liquid state. The resulting magnetic and electric anisotropies of NLC molecules allow their orientation in response to a magnetic or electric field. This phenomenon can lead to a variety of interesting applications. Additionally, the usage of dopants represents another way to expand the application of NLCs. Doped NLCs can contribute to the development of novel materials based on the controlled arrangement of particles under applied fields. However, the properties of such materials depend on several experimental parameters, particularly on the characteristics of the nanoparticles, such as their concentration, size, and shape, as well as on NLC cell surface treatment and the applied electric or magnetic fields. The properties of doped NLCs can differ significantly from those of the host material, and in this way, doping can enhance the NLCs with remarkable new properties compared to the pure material [1,2,3,4]. Thus, the combination of the anisotropy of NLCs with the unique properties of nanoparticles can create new perspective NLC composites, the properties of which can be modified by external stimuli such as magnetic or electric fields, temperature, or light, producing new materials suitable for nanotechnology development [5,6,7,8,9].

NLCs doped with magnetic nanoparticles represent magnetically active anisotropic fluids, suggesting that doping NLCs with suitable magnetic nanoparticles can enhance their sensitivity to magnetic fields [10,11,12,13,14]. The key attribute of such composites is the coupling between the average magnetic moment ***m*** of the nanoparticles and the director ***n***, which is characterized by the preferred orientation of nematic molecules. Under an applied magnetic field, this coupling can lead to the reorientation of the NLC molecules. The resulting improvement in sensitivity to the magnetic field can influence their characteristics, including threshold field, phase transition, electro-optical and magneto-optical behavior, etc. [15,16,17,18].

Transition metal ferrite nanoparticles, due to their unique properties that make them appropriate for application in various fields such as ferrofluids, magnetic storage, magnetic refrigeration systems, etc., can represent one group of suitable magnetic nanoparticles [19,20,21,22,23]. Zinc ferrite (ZnFe_2_O_4_) nanoparticles, belonging to this group of materials, exhibit unique electrical, magnetic, magneto-optical, magneto-resistive, thermal, and optical properties, suggesting strong potential for technological applications [19,22,23]. Therefore, a composite of NLC and ZnFe_2_O_4_ nanoparticles could be considered for applications in LC-based devices. Recent results on the dielectric and electro-optical properties of ZnFe_2_O_4_ nanoparticles dispersed in nematic 7CB [24] at different concentrations (from 0.05 to 0.5 wt%) indicate uniform nanoparticle dispersion at low concentrations and demonstrate a significant influence of nanoparticle concentration on the dielectric and electro-optical properties. Regarding the memory effect, while only a small memory was observed in the pure NLC, a noticeable memory was found in the 7CB/ZnFe_2_O_4_ composites, again depending on the nanoparticle concentration. The effects of zinc ferrite nanoparticles dispersed in PCH5 on dielectric and selected optical characteristics [25] confirmed the significant influence of both nanoparticle concentration in the NLC and the applied electric field on the observed results. Zeta potential measurements provided information about stability and aggregation dynamics in the studied composites. In summary, ZnFe_2_O_4_ nanoparticle–dispersed NLC systems may be of interest due to their ability to modify the properties of the host NLCs, enabling wide applications in NLC-based devices.

Results obtained on nickel zinc ferrite (Ni_0.5_Zn_0.5_Fe_2_O_4_) dispersed in chiral NLC have shown once more that these nanoparticles can significantly influence physical parameters such as dielectric properties, threshold voltage, or elastic constants of NLC [26]. Threshold voltage and splay elastic constant have been decreased, and photoluminescence intensity has increased with the presence of a fitting concentration of NiZnFe_2_O_4_ nanoparticles. Nickel zinc ferrite (Ni_0.5_Zn_0.5_Fe_2_O_4_) nanoparticles are a type of magnetic nanoparticles characterized by low magnetic and dielectric loss and with a high value of magnetization. Concerning its inner structure, NiZnFe_2_O_4,_ consisting of nonmagnetic Zn^2+^ at the tetrahedral site and magnetic Ni^2+^ at the octahedral site, is a mixed spinel with iron atoms in each site [27,28]. A strong interaction between guest nickel zinc ferrite nanoparticles and host chiral NLC molecules in this composite structure could make the material promising for optoelectronic applications.

The Mn^2+^ ions in MnZnFe_2_O_4_ nanoparticles play, similarly to nickel in zinc ferrite, a decisive role in determining the magnetic properties of Mn–Zn-ferrite nanoparticles. The substitutions of Mn^2+^ ions influence, in this case, the degree of inversion, magnetic moment of MnZnFe_2_O_4_ particles, and the transition temperature between antiferrimagnetic and paramagnetic states [29,30]. Substituting Mn in Zn-ferrite, the ferromagnetic interactions will be preferred. The replacement of Zn by 50% Mn (Mn_0.5_Zn_0.5_Fe_2_O_4_) results in an increase in ferromagnetic interactions with no significant change in the crystallite size [31]. Recently, Mn_0.5_Zn_0.5_Fe_2_O_4_ particles of suitable shape were used in a composition that has the potential to function as a temperature-controlled magnetic switch for magnetic fluid hyperthermia [32].

This contribution presents the first investigation into the effect of Mn-doped ZnFe_2_O_4_ nanoparticles, a material of growing research interest, on structural transformations induced by a magnetic field and on the magneto-optical properties of composites based on the nematic liquid crystals 5CB and 6CHBT. Structural changes were investigated using measurements of both surface acoustic wave (SAW) attenuation and light transmission (LT) responses to obtain information about the behavior of the studied NLC composites under an external magnetic field, with the main aim of assessing the influence of MnZnFe_2_O_4_ nanoparticles as a new dopant on the structural and magneto-optical properties of NLCs, which have not been previously investigated. The combination of the utilized experimental techniques, the results of which are presented here, has already proven to be very effective [33,34].

## 2. Materials and Methods

Mn_0.5_Zn_0.5_Fe_2_O_4_ samples with rod-like, clustered, and needle-like morphologies were synthesized using hydrothermal and solvothermal techniques. In the preparation, the rods and needle samples each employed a combination of FeSO_4_·7H_2_O, MnSO_4_·H_2_O, and ZnSO_4_·7H_2_O in a 2.0:0.5:0.5 molar ratio. For rods, 4.5 mM of metal sulfates were dissolved in 12 mL of a 3 mM KNO_3_ aqueous solution, then 12 mL of 2 M NaOH and 96 mL of a 100-fold diluted PEI solution were added, resulting in a highly alkaline mixture (with a pH of 12.95) that was heated at 100 °C for 12 h. In contrast, needle synthesis was performed by dissolving 6 mM of metal salts in a 110 mL water–benzene medium with the addition of 20 mL of ethylenediamine (EDA) as a coordinating agent, and the mixture was subjected to a higher reaction temperature of 150 °C for 30 h. The synthesis procedure was followed by magnetic separation and washing with distilled water to remove impurities. Whereas clusters were synthesized by taking the molar ratio of Mn:Zn:Fe precursor with 0.5:0.5:2.0 of MnCl_2_·4H_2_O, ZnCl_2_, and FeCl_3_·6H_2_O. The 14.8 mM of salt and 0.175 M of NaAc precipitating agent were added to 160 mL of ethylene glycol. The mixture was stirred for 30 min at room temperature and then sealed into a Teflon-lined autoclave vessel, where it was heated for 16 h at 200 °C. The resultant product was washed with distilled water.

Concerning the purity of used compounds, iron (III) chloride hexahydrate (FeCl_3_·6H_2_O(s), 98%), manganese (II) chloride tetrahydrate (MnCl_2_·4H_2_O(s), 98%), tetramethylammonium hydroxide (TMAOH, N(CH_3_)_4_ OH, ≥98%) manganese (II) sulfate monohydrate (MnSO_4_·H_2_O(s), >99%, 169.02 g/mol), zinc sulfate heptahydrate (ZnSO_4_·7H_2_O(s), 99%, 287.56 g/mol), and poly(ethyleneimine) solution (PEI, 1800 g/mol, 50 wt% in water) were purchased from Sigma Aldrich (St. Louis, MO, USA). Sodium acetate trihydrate (CH_3_COONa·3H_2_O, 99.5%) and ethylenediamine (EDA, >99%) were purchased from Merck (Burlington, MA, USA). Ethylene glycol (99%), potassium nitrate (KNO_3_, 99%), and sodium hydroxide (NaOH, 98%) were from Samir Tech-Chem. Pvt Ltd. (Vadodara, Gujarat). India Ferrous sulfate heptahydrate (FeSO_4_·7H_2_O(s), 99%) was purchased from HIMEDIA (Thane, India), and zinc (II) chloride (ZnCl_2_(dry), 98%) and benzene (C_6_H_6_, 98%) were purchased from LOBA Chemie Pvt. Ltd., Mumbai, India. All these chemicals were used without any additional purification.

Samples of NLC were based on the thermotropic nematic 4-(trans-40-n-hexylcyclohexyl)-isothiocyanatobenzene (6CHBT), which has high chemical stability and low viscosity, and on the nematic LC 4-cyano-4′-pentylbiphenyl (5CB) that was purchased from the Military University of Technology (MUT, Warsaw, Poland). The nematic liquid crystal samples were prepared using two thermotropic NLCs: 4-(trans-4′-n-hexylcyclohexyl)-isothiocyanatobenzene (6CHBT), known for its high chemical stability and low viscosity, and 4-cyano-4′-pentylbiphenyl (5CB). Both materials were obtained from the Military University of Technology (MUT, Warsaw, Poland). The composites were doped with MnZnFe_2_O_4_ nanoparticles at a volume concentration of 1 × 10^−4^, using three nanoparticle morphologies (rods, clusters, and needles). To achieve homogeneous dispersion, the NLC–nanoparticle mixtures (Mn_0.5_Zn_0.5_Fe_2_O_4_) were heated above their isotropic temperatures (40 °C or 50 °C) and subjected to ultrasonic mixing for 2 h. All nanoparticles used in the preparation were uncoated powders.

The magnetic properties of the powder samples were investigated using a vibrating sample magnetometer (VSM) installed on a cryogen-free superconducting magnet from Cryogenic Limited (UK). The VSM operates in a temperature range from 2 K to 320 K and a magnetic field range from 0 to 18 T. The magnetic moment sensitivity of VSM is 10^−6^ emu. Before the measurements, the VSM was calibrated with a yttrium iron garnet standard sample. A small amount of powder (a few mg) was inserted into a gelatin capsule. The mass of the powder was measured using a precise scale. The powder in the capsule was fixed with parafilm. Finally, the capsule with the powder was mounted on the vibrating rod of the VSM, and the magnetic moment was measured. Then, the specific magnetization of the powder was calculated as the ratio of the measured magnetic moment and the sample mass. The magnetization results of the three distinct nanostructured samples (rods, clusters, and needles) were obtained through field-dependent magnetization (M-H) and temperature-dependent magnetization (M-T) measurements. Each sample was measured in one capsule. However, the magnetization was measured three times at each temperature and magnetic field value, yielding reproducible results for the sample in one capsule.

**Figure 1:**
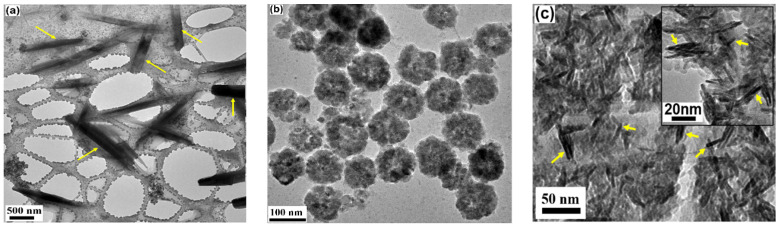
TEM images of MnZn-ferrite in rods (**a**), clusters (**b**), and needles (**c**) shapes. Rods and needles are marked with arrows.

The morphological characterization and particle size of the samples were determined by analyzing TEM images. Figure 1 displays the TEM images of all three samples: (a) rods, (b) clusters, and (c) needles. The length and diameter of the rod and needle samples, as well as the size of the clusters, were measured using ImageJ software (https://imagej.net/ij/) and fitted with a lognormal distribution function. The length obtained for the rods was 1883.2 ± 600 nm, while for needles it was 22.9 ± 0.4 nm. The diameter of the rods and needles, as determined from the fit, was 357.2 ± 26 nm and 3.1 ± 0.04 nm, respectively. The size of the clusters found was 106.8 ± 1.3 nm.

**Figure 2:**
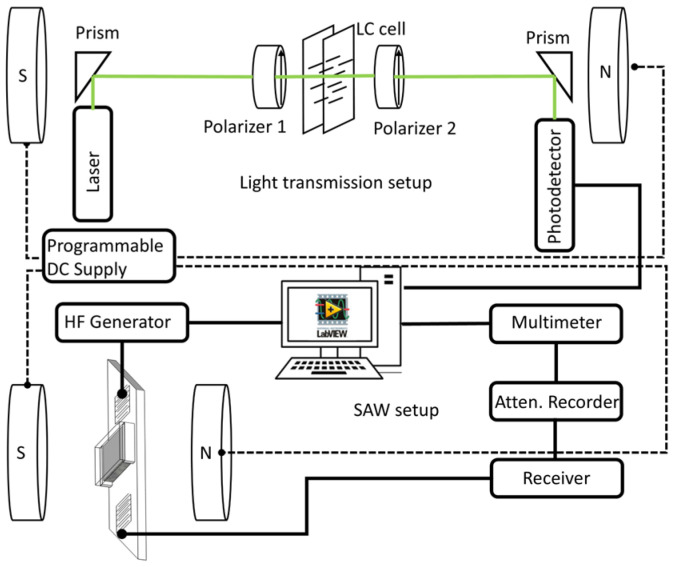
Schematic experimental arrangement of LT (upper part) and SAW (lower part) measurements.

To examine the influence of MnZnFe_2_O_4_ nanoparticles of different shapes and different sizes on the structural changes in NLCs (6CHBT and 5CB) under a weak magnetic field, LT experiments were conducted in cells with a 50 μm cell gap. Cells for NLC composites were coated with ITO transparent conductive layers, and alignment layers were rubbed in the parallel direction, considering the electrodes. A linearly polarized laser beam (532 nm) illuminated the cell’s glass surface at normal incidence (Figure 2—upper part). The transmitted light intensity from the NLC cell was recorded using a photodetector linked to a computer, enabling continuous monitoring of LT as a function of the magnetic field or time. The LT was expressed as *I/I*_0_ for parallel polarizers, where *I*_0_ is the maximal intensity of incident light passing through the NLC cell, and *I* represents the running light intensity under the applied field. No bias field was imposed to investigate suspensions.

The cells for SAW measurements containing the NLC composites with a thickness of ~100 μm were prepared directly at the center of the LiNbO_3_ substrate between two interdigital transducers (Figure 2—lower part). The substrate holder was part of the thermostatic measuring chamber. SAW pulses (~1 µs) at a frequency of 10 MHz were generated by the first interdigital transducer and, after passing the NLC cell, were registered by the second transducer. The received signal was detected using the attenuation recorder, and similarly, the LT intensity was monitored as a function of the magnetic field or time. The initial intrinsic arrangement of NLC molecules in the measuring SAW cell is supposed to have a predominant alignment in the plane of the NLC cell. The reason is that we used an optically polished LiNbO_3_ delay line and covered glass. Thus, due to the anchoring energy of LC molecules, we can suppose planar alignment. Magnetic field was, similarly to the case of LT measurements, applied perpendicular to the cells. The instability for the SAW attenuation changes Δ*α* measurement was smaller than ±0.02 dB, and temperature could be stabilized in the range of 5–80 °C with an accuracy of ±0.2 °C.

## 3. Results

**Figure 3:**
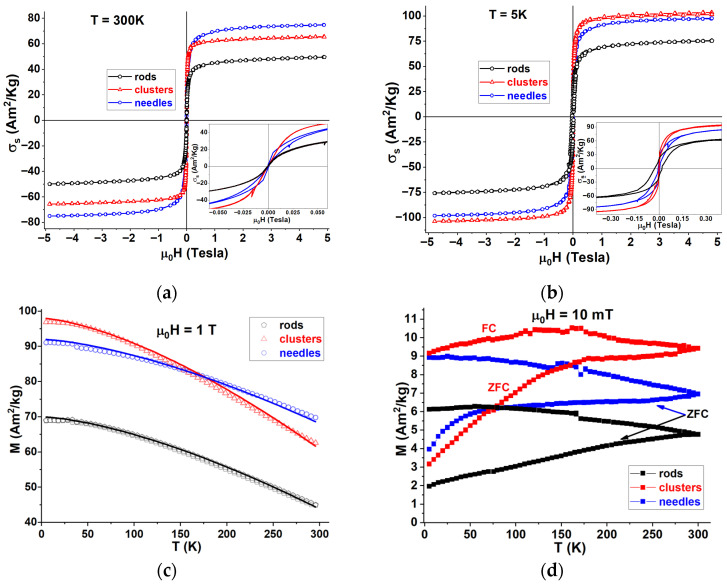
Magnetic characterizations of rods, clusters, and needles (**a**) M–H curves at 300 K, (**b**) M–H curves at 5 K, (**c**) M–T curves at a magnetic field of 1 T (scattered plot) fitted with the Bloch law (solid line), and (**d**) ZFC—FC curves of all three samples.

Magnetic characterization of MnZnFe_2_O_4_ nanoparticles using M-H curves at 300 K, measured in the magnetic field range from −5 to 5 T, as shown in Figure 3a, exhibits a typical superparamagnetic behavior for all samples. The resultant saturation magnetization obtained for rods, clusters, and needles is 51.6 Am^2^/kg, 67.4 Am^2^/kg, and 76.5 Am^2^/kg, respectively. Figure 3b represents the magnetic hysteresis loops at 5 K in the same magnetic field range from −5 T to 5 T. Moreover, the insert of Figure 3b displays the low-field magnetic response, showing the coercivity and remanence present at low temperature, and reveals a ferrimagnetic nature of all the samples. The revealed coercivity is 0.0054, 0.0015, and 0.0227 T for needles, clusters, and rods, respectively. The rods exhibit the greatest remanence, 21.1 Am^2^/kg, while 19.3 and 11.6 Am^2^/kg are found for needles and clusters, respectively. The resultant saturation magnetization attained is 78.2 Am^2^/kg, 105.4 Am^2^/kg, and 99.4 Am^2^/kg for rods, clusters, and needles, respectively. It shows constricted hysteresis, which indicates the possibility of exchange bias, spin canting, and surface effects. Figure 3c represents the temperature-dependent magnetization recorded at a 1 T magnetic field between the temperature range of 5 K to 300 K (scattered plot). The data are fitted using the Bloch law [35] to obtain the Curie temperature of the synthesized rods, clusters, and needle samples (solid line). Equation (1) is used to fit the temperature-dependent magnetization data.

(1) MsT=Ms0 1−TTC32

Here, *M_s_*(0) represents the spontaneous saturation magnetization at 0 K, and *T_C_* denotes the Curie temperature. The fitted plots are shown in Figure 3c by a solid line through the data points. The Curie temperature obtained from the fit for the rods, clusters, and needles is 577.8 K, 572.5 K, and 736.8 K, respectively.

**Figure 4:**
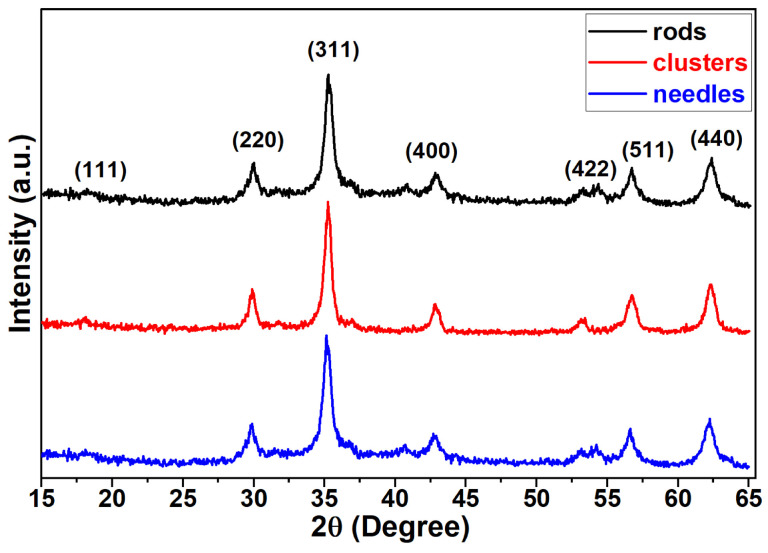
XRD pattern of rods, clusters, and needle shapes.

In Figure 3d, one observes the temperature-dependent magnetization behavior measured in zero-field-cooling (ZFC) and field-cooling (FC) regimes, with the measuring magnetic field set to 10 mT. The ZFC magnetization curves (lower curves) constitute a continuous increase with temperature. A remarkable ZFC shoulder is observed for the magnetization of needles, with a maximum around 60 K. The shoulder indicates the area of blocking temperatures causing the magnetization transition from the superparamagnetic to the blocked state. An expanded ZFC shoulder is found for clusters with a maximum close to 180 K. The expansion reflects a broader size distribution of the clusters. On the other hand, the ZFC magnetization of rods does not exhibit any maximum, which, in view of the shape anisotropy of the nanoparticles, may reflect the dipolar and exchange interactions between the rod-like nanoparticles. The FC magnetization curves found for rods and needles increase with decreasing temperature, while the FC curve of clusters shows a maximum around 150 K. The powder XRD pattern of rods, clusters, and needle samples is shown in Figure 4. The resultant pattern confirms the presence of the face-centered cubic (FCC) structure with an Fd-3m space group, which exhibits an inverse spinel structure. The crystallite size and lattice parameters of the samples were determined using MAUD refinement software (https://luttero.github.io/maud/). The crystallite sizes of the rods, clusters, and needles, after refinement, were found to be 13.1 ± 0.2 nm, 19.2 ± 0.4 nm, and 14.2 ± 0.7 nm, respectively, which is about twice as large as previously measured in powder form [31]. The lattice parameters were 0.8429 ± 0.0004 nm, 0.8419 ± 0.0004 nm, and 0.8437 ± 0.0007 nm, respectively. The typical reported lattice parameter for the Mn_0.5_Zn_0.5_Fe_2_O_4_ bulk system is 0.8421 nm, and the obtained lattice constant values of all the samples are very close to the reported value of the bulk system.

**Figure 5:**
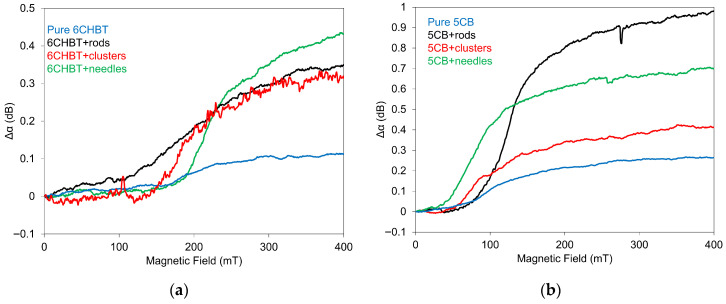
Effect of applied magnetic field on SAW attenuation for 6CHBT (**a**) and 5CB (**b**) liquid crystals doped with Mn–Zn-ferrite in rod, cluster, and needle shapes, including pure NLCs.

Structural changes in NLC composites doped with Mn–Zn-ferrite nanoparticles (Mn_0.5_Zn_0.5_Fe_2_O_4)_ under applied magnetic fields were monitored by measuring the attenuation *α* of SAW propagating along the interface between the LiNbO_3_ delay line and the NLC cell. As demonstrated in our previous studies [35,36,37], the interaction between the magnetic moments ***m*** of the nanoparticles and the director ***n*** promotes an initially parallel alignment of the NLC molecules with the magnetic moments. When a magnetic field perpendicular to the NLC cells was applied, the director reoriented toward the field direction, causing the NLC molecules to rotate toward a perpendicular orientation with respect to the electrode surfaces. The SAW attenuation consequently changed in response to these reorientation processes. Figure 5 shows the effect of the applied magnetic field on the SAW attenuation change in 6CHBT (a) and 5CB (b) doped with Mn–Zn-ferrite nanoparticles of the same concentration but different shapes. The concentration 1 × 10^−4^ was chosen as an adequate concentration for the comparison of the role of different nanoparticle shapes. The magnitude of the magnetic field effect depends on both the shape of the magnetic particles and the type of NLC. In nearly all investigated composites, the attenuation changes (Δ*α*) associated with structural alterations in a magnetic field displayed a similar and characteristic pattern [33,36], characterized by a weak initial increase in the ranges of 0–150 mT and 0–70 mT, respectively, followed by a steeper rise that gradually leveled off toward saturation.

It is evident that in all composites, regardless of the NLC type or the shape of the nanoparticles, the changes in SAW attenuation, and thus the corresponding structural changes, are larger than in the pure NLCs. This confirms the role of Mn–Zn-ferrite nanoparticles as magnetically active components. Interestingly, the increase in attenuation Δα in 5CB-based composites is more than twice that observed in the 6CHBT composites. This difference reflects the distinct magnetic properties of the molecules forming the two pure NLCs, which is also evident from their behavior in a magnetic field (see the curves for the pure NLCs in Figure 5). However, the lower viscosity of 6CHBT compared to 5CB may also contribute to the observed behavior.

The important feature of individual nanoparticle types, concerning their shape, that should be mentioned is their size, which is significantly different. This varies from ~23 nm (needles) through ~106 nm (clusters) up to ~1900 nm (rods). Concerning the threshold field, it is evident that MnZnFe_2_O_4_ nanoparticles shift the threshold field in both NLCs, 6CHBT and 5CB, compared with pure ones. However, the magnitude and direction of this shift depend strongly on the nanoparticle shape. 6CHBT composite with nanoparticles of rod shape showed the largest decrease in the threshold field relative to both the pure NLC and the other doped 6CHBT samples. The remaining two nanoparticle types caused weaker shifts, where clusters lowered the threshold field slightly, while needles shifted it to higher values compared to pure 6CHBT. Similar situations can be observed in 5CB composites. However, the largest reduction in the threshold field was observed in the composite containing needle-shaped nanoparticles. Nanoparticles with elongated shapes, such as rods or needles, allow their magnetic moments to reorient more easily in the direction of the applied magnetic field compared to cluster-type particles. The obtained results are consistent with earlier findings [38]. These observations clearly indicate that, when doped with MnZnFe_2_O_4_ nanoparticles, NLC molecules can be reoriented not only by an electric field but also by a magnetic field. The obtained results confirm the influence of nanoparticle shape, and consequently size, on the magnetic threshold field, as well as on the subsequent evolution of structural changes. The position of the threshold field is affected by the presence of ferroparticles and arises from a combination of their ferromagnetic and anchoring energies, both of which depend on nanoparticle geometry. The shift in the threshold magnetic field toward lower values indicates a preferred parallel alignment of the magnetization vector with the liquid crystal director. Regarding the two nanoparticle shapes (rods and clusters), only minor changes in the threshold field were observed compared with pure 5CB. In contrast, the composite containing needle-shaped nanoparticles exhibited a pronounced decrease in the threshold field relative to pure 5CB.

For 6CHBT, the composites doped with nanorod-shaped particles showed the largest reduction in the threshold field among all the 6CHBT systems investigated. The other two nanoparticle types produced smaller shifts: clusters led to a slightly lower threshold field, while needles resulted in a slightly higher value compared with pure 6CHBT. Capacitance measurements were used to calculate the surface anchoring energy density W and the anchoring parameter ω, indicating rigid anchoring for this type of NLC with rod-shaped nanoparticles [38]. The important role of investigated composites could denote the different sizes of individual nanoparticle types [38]. This fact could also be the reason for some ambiguous behavior.

**Figure 6:**
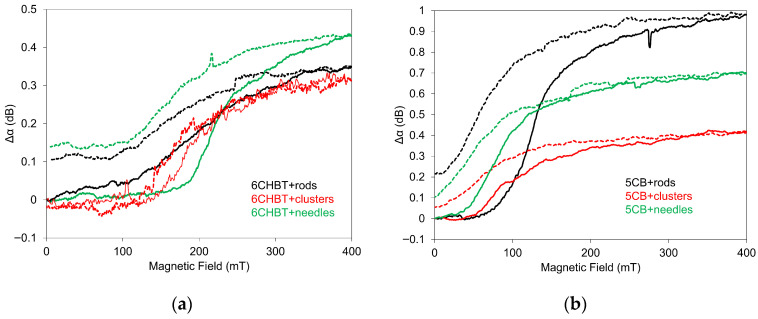
Memory effect after applied increasing (solid lines) and decreasing (dashed lines) magnetic field registered by SAW attenuation for 6CHBT (**a**) and 5CB (**b**) liquid crystals doped with Mn–Zn-ferrite in rod, cluster, and needle shapes.

In comparison with Figure 5, Figure 6 illustrates the influence of an applied magnetic field on SAW attenuation during the decreasing field regime for 6CHBT (a) and 5CB (b) composites doped with MnZnFe_2_O_4_ particles of various shapes: rod, cluster, and needle. A relatively large memory effect was observed in all investigated composites, representing ~15–20% of maximal changes. These residual SAW attenuation values are comparable to those reported for zinc-ferrite nanoparticles dispersed in nematic 7CB [24], although in that case the effect was induced by an electric field. The reproducibility in our measurements was confirmed in the sense that every new cell prepared from the NLC composite shows the same results. However, when measurements were repeated in the increasing and subsequently decreasing field regimes, noticeable differences appeared. These discrepancies arise from persistent structural changes within the composites, which remain after the first application of the field.

**Figure 7:**
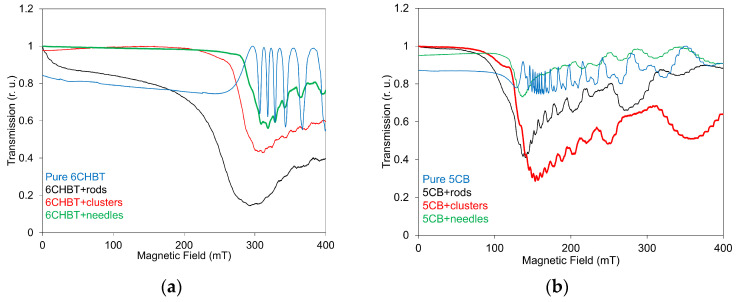
The dependences of light transmission on increasing magnetic field for 6CHBT (**a**) and 5CB (**b**) liquid crystals doped with MnZnFe_2_O_4_ in rod, cluster, and needle shapes, including pure NLCs.

LT measurements were performed on the same set of NLC samples doped with Mn–zinc ferrite nanoparticles, using the experimental arrangement described in the previous paragraph. The influence of the magnetic field on LT for both 6CHBT and 5CB composites, each containing MnZnFe_2_O_4_ nanoparticles of identical concentration but different shapes, is presented in Figure 7. For the 6CHBT composites (Figure 7a), an initial, nearly linear decrease in LT is observed up to approximately 200–270 mT. Beyond this range, a much faster drop in transmission occurs, followed by a gradual increase toward a saturation state. During this final stage, slightly superimposed oscillations can be detected. Notably, in the composites containing rod-shaped nanoparticles, the LT begins to change immediately after the magnetic field is applied. Such development can be attributed to the presence of different regimes of threshold orientational behavior under a magnetic field characteristic for compensated ferronematics [39,40]. The reason for this behavior is attributed to the gradual magnetization of the ferronematic in the director orientation, rotation of the director in the external field, and finally synchronous rotation of the director and magnetization along the applied field. A similar trend in magneto-optical characteristics was also observed in the case of 6CHBT doped with functionalized SWCNTs [33] or rod magnetic nanoparticles [39]. The shift in the threshold field is consistent with the results obtained from the SAW measurements. Similar behavior of the light transmission was observed for the 5CB composites doped with MnZnFe_2_O_4_ nanoparticles (Figure 7b); although the threshold field was lower (~100–140 mT), the corresponding shift was less pronounced, and the superimposed oscillations were more distinct. The different values of the threshold field in LT characteristics, with respect to SAW measurements, can be attributed to the fact that the threshold field *B_c_* depends on the NLC cell thickness *D* (*B_c_* ~ 1/*D*) [37].

The comparison of obtained characteristics confirms the fact, already determined from SAW measurements, that different sensitivity to the magnetic field can be registered depending on the nanoparticles’ shape, which can also be influenced by the nanoparticles’ size. Although composites containing rod-shaped nanoparticles exhibit changes in LT immediately after the magnetic field is applied, the overall magnitude of LT changes is determined by the nanoparticle shape. It is evident that the influence of the magnetic field on LT decreases progressively from composites with rod-shaped nanoparticles to those with needle-shaped ones, where the effect on LT in the host liquid crystal becomes minimal. Comparing the results of LT measurements with the earlier SAW attenuation data, the influence of nanoparticle shape on the structural changes in the investigated composites may initially appear different. SAW measurements indicate that rod- or needle-shaped nanoparticles allow their magnetic moments to reorient more easily along the direction of the applied magnetic field than cluster-shaped particles, resulting in more pronounced structural changes. An additional factor relevant for LT measurements is the notably smaller size of the needle nanoparticles compared with the other two types.

**Figure 8:**
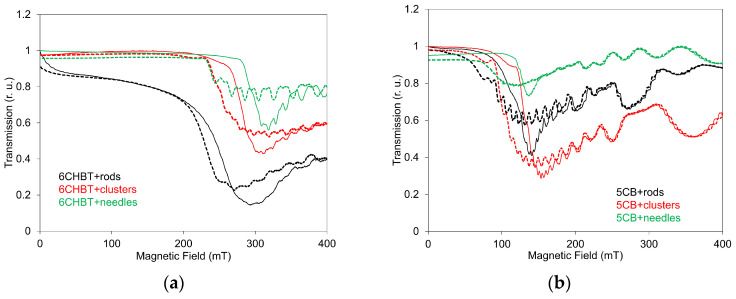
Dependences of light transmission on increasing (solid lines) and decreasing (dashed lines) magnetic field for 6CHBT (**a**) and 5CB (**b**) liquid crystals doped with MnZnFe_2_O_4_ in rod, cluster, and needle shapes.

However, certain differences between the two methods can also arise from the intrinsic differences in the information each technique provides. SAW measurements predominantly probe structural changes in the NLC layer directly adjacent to the LiNbO_3_ piezoelectric substrate and therefore reflect modifications occurring near this interface. In contrast, LT measurements are sensitive to structural changes integrated across the full thickness of the NLC cell, but only within the confined region defined by the laser beam’s cross-section. Consequently, each method highlights different spatial aspects of field-induced structural reorganization. In LT measurements, changes occurring within smaller, localized regions can appear more pronounced than the bulk reorientational processes detected by SAW measurements. LT data recorded during the decreasing magnetic-field regime for both 6CHBT and 5CB composites (Figure 8) reveal the presence of memory effects in all examined systems, amounting to approximately 5–20% of the maximum LT variation, depending on nanoparticle shape and the type of NLC. However, these memory effects are significantly weaker than those observed in the SAW measurements. This indicates that certain structural changes detected by SAW attenuation do not substantially influence the optical response of the composites.

**Figure 9:**
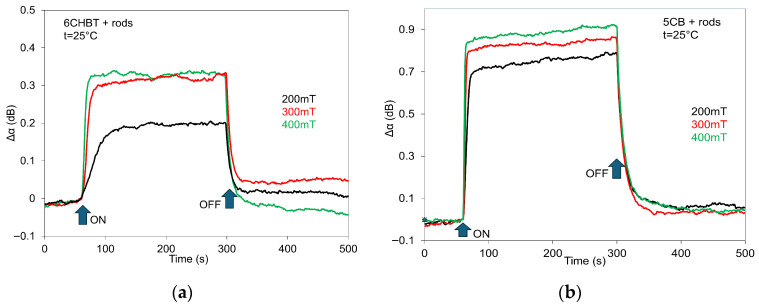
Effects of applied magnetic field (200, 300, and 400 mT) on SAW attenuation for 6CHBT (**a**) and 5CB (**b**) doped with MnZnFe_2_O_4_ nanoparticles of the same shape (rods).

**Figure 10:**
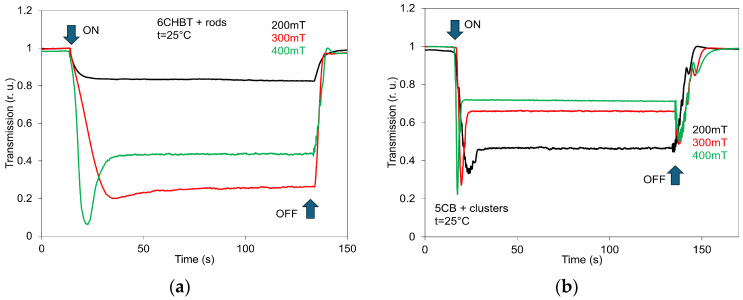
LT time responses of 6CHBT liquid crystals doped with MnZnFe_2_O_4_ nanoparticles in rods (**a**), and 5CB with cluster shape (**b**) for different magnetic fields (200, 300, and 400 mT) as representative results of the switching process.

The evolution of the switching process and the influence of different magnetic field strengths (200, 300, and 400 mT) on SAW attenuation in 6CHBT (a) and 5CB (b) doped with MnZnFe_2_O_4_ nanoparticles of the same shape (rods), shown as representative examples, are presented in Figure 9. The time constants of the switching processes reflect the structural changes triggered by a pulsed variation in the applied magnetic field. These changes arise from the response of the magnetic moments of MnZnFe_2_O_4_ nanoparticles and their interaction with the liquid-crystal director. However, relaxation times also depend on the magnitude of the magnetic field, which is clearly visible in the time responses recorded for the three applied field strengths. This behavior is consistent with previous magneto-optical and electro-optical studies [3,39]. The obtained results confirmed that the rise and delay switching times are also influenced by the shape of the nanoparticles [39]. Therefore, by appropriately selecting not only their shape but also their size and concentration, it is possible to design an NLC suspension with tailored properties. The presented time responses, similarly to those observed in other composites, are consistent with the behavior of the SAW attenuation characteristics (Figure 5). However, due to the memory effect observed in the SAW attenuation responses (Figure 6), the amplitudes of the SAW jumps are slightly lower than what would be expected based on the attenuation characteristics alone. Figure 10 presents the LT time responses (switching processes) for 6CHBT liquid crystals doped with MnZnFe_2_O_4_ nanoparticles in rod form (a) and for 5CB doped with cluster-shaped nanoparticles (b), recorded at different magnetic field strengths (200, 300, and 400 mT) as representative examples. The LT time responses, similar to those obtained for the other composites, are fully consistent with the corresponding LT characteristics shown in Figure 7a and Figure 8b. This indicates that the LT signal relatively quickly overcomes any previous state and stabilizes at the appropriate level determined by the applied magnetic field.

**Figure 11:**
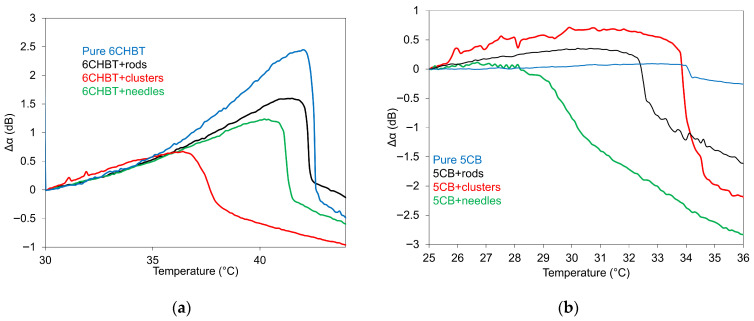
Temperature dependence of SAW attenuation for 6CHBT (**a**) and 5CB (**b**) liquid crystals doped with Mn–Zn-ferrite in rod, cluster, and needle shapes of nanoparticles, including pure NLC.

The temperature dependences of acoustic attenuation for 6CHBT (a) and 5CB (b) liquid crystals doped with MnZnFe_2_O_4_ nanoparticles of rod, cluster, and needle shapes at a volume concentration of *Φ* = 10^−4^, as well as for pure NLCs, are illustrated in Figure 11. The SAW attenuation with increasing temperature (0.2 °C/min.) shows, in all composites, an initial slow, gradual increase followed by a more pronounced decrease, which corresponds to the nematic–isotropic transition (*T_NI_*). In some composites, the moderate drop in SAW attenuation is followed by a slower, additional decrease with further temperature increase, indicating the transition to a paranematic state [41]. Concerning the transition temperatures *T_NI_* of both kinds of NLC composites, their decrease concerning pure ones was registered in order of rods, needles, and clusters in the case of 6CHBT composites (Figure 11a), whereas for 5CB composites (Figure 11b), it is needles, rods, and clusters, with a particularly pronounced shift for composites containing cluster nanoparticles. The shift in phase transition towards lower temperatures for NLC composites can be attributed to the anchoring interactions between NLC molecules and nanoparticle surfaces, which disrupt the orientational order of the NLC [42]. The typical example could be a composition of 6CHBT and cluster nanoparticles. A typical example is the 6CHBT composite with cluster nanoparticles. Another factor contributing to the decrease in the nematic–isotropic transition temperature could be the increase in impurity volume generated during nanoparticle synthesis [43].

A similar shift in phase transition towards lower temperatures was observed for NLCs doped with Zn-ferrite nanoparticles [26], measured even for several different concentrations. The development of SAW attenuation as a function of temperature also indicates the role of thermal motion on the structural changes detected through the interaction of surface acoustic waves with LC molecules.

## 4. Conclusions

In this paper, we present for the first time a study of the role of MnZnFe_2_O_4_ nanoparticles in structural changes in NLCs, 6CHBT, and 5CB induced by weak magnetic fields. Mn-doped zinc ferrite nanoparticles of different shapes (rods, needles, and clusters) and various sizes within each type, mixed with NLCs at the same concentration (1 × 10^−4^), were prepared for investigation using SAW and light transmission. Structural changes induced by the applied magnetic field, in both increasing/decreasing modes as well as after stepwise changes, were investigated by measuring the responses of SAW attenuation and LT using a linearly polarized laser beam. In addition, the magnetic properties of individual samples in powder form were investigated using M–H curves, M–T curves, and XRD patterns. The shift in threshold fields and transition temperature compared to pure NLCs was registered. However, depending on the nanoparticle shape, the size of individual nanoparticle types was an important factor influencing the results. The findings consistently highlighted the role of both shape and size in determining structural changes in NLC composites under an external field. Investigation of the switching processes confirmed that the reorientation rate of NLC molecules depends on the strength of the applied external field and that the progression of switching is a function of field intensity. The presented magneto-optical results match our previous findings on the same NLCs doped with different nanoparticles. Doping NLCs with MnZnFe_2_O_4_ nanoparticles demonstrated that, due to effective orientational coupling between the nanoparticles’ magnetic moments and the NLC director, this type of zinc ferrite can serve as an active dopant in magnetically responsive NLC suspensions. The obtained results support previous conclusions that the shape and size of magnetic nanoparticles incorporated into the host NLC play a critical role in the stability of its magneto-optical properties. In summary, these findings indicate that the integration of MnZnFe_2_O_4_ nanoparticles into NLCs can enhance both the magnetic properties of the host suspension and its magneto-optical behavior, making it suitable for the development of novel materials for various applications, such as magnetic sensors, smart systems, and magnetic storage devices.

## Data Availability

The original contributions presented in this study are included in the article. Further inquiries can be directed to the corresponding author.

## Abbreviations

The following abbreviations are used in this manuscript:

SAW	Surface Acoustic Wave
LT	Light Transmission
NLC	Nematic Liquid Crystal
ITO	Indium Tin Oxide
ZFC	Zero-field-cooling
FC	Field-cooling
XRD	X-ray diffraction
